# Functional Organization of Locomotor Interneurons in the Ventral Lumbar Spinal Cord of the Newborn Rat

**DOI:** 10.1371/journal.pone.0020529

**Published:** 2011-06-17

**Authors:** Myriam Antri, Nicholas Mellen, Jean-René Cazalets

**Affiliations:** 1 Université de Bordeaux, Centre National de la Recherche Scientifique, Institut des Neurosciences Cognitives et Intégratives d'Aquitaine, Unité Mixte de Recherche 5287, Bordeaux, France; 2 Kosair Children's Hospital Research Institute, University of Louisville, Louisville, Kentucky, United States of America; Emory University, United States of America

## Abstract

Although the mammalian locomotor CPG has been localized to the lumbar spinal cord, the functional-anatomical organization of flexor and extensor interneurons has not been characterized. Here, we tested the hypothesis that flexor and extensor interneuronal networks for walking are physically segregated in the lumbar spinal cord. For this purpose, we performed optical recordings and lesion experiments from a horizontally sectioned lumbar spinal cord isolated from neonate rats. This ventral hemi spinal cord preparation produces well-organized fictive locomotion when superfused with 5-HT/NMDA. The dorsal surface of the preparation was visualized using the Ca^2+^ indicator fluo-4 AM, while simultaneously monitoring motor output at ventral roots L2 and L5. Using calcium imaging, we provided a general mapping view of the interneurons that maintained a stable phase relationship with motor output. We showed that the dorsal surface of L1 segment contains a higher density of locomotor rhythmic cells than the other segments. Moreover, L1 segment lesioning induced the most important changes in the locomotor activity in comparison with lesions at the T13 or L2 segments. However, no lesions led to selective disruption of either flexor or extensor output. In addition, this study found no evidence of functional parcellation of locomotor interneurons into flexor and extensor pools at the dorsal-ventral midline of the lumbar spinal cord of the rat.

## Introduction

To understand how the neural networks implicated in locomotion might work, it is of great importance to identify their constituents and also to determine their spatial organization [1]. In neonatal rat, numerous studies have characterized putative neurons involved in rhythmic locomotor behaviour. Intracellular recordings have been used to study the cellular properties of unidentified spinal interneuron populations [2], [3], [4], [5] and identified interneurons, such as commissural interneurons involved in left/right coordination [6], [7], [8]. More recently, molecular biological techniques have permitted a systematic classification of diverse ventral spinal cord (SC) interneuronal cell types hypothesized to be constituents of the mammalian locomotor CPG [9], [10] such as Ephrine-4 positive interneurons [11], Hb9 positive interneurons [12] and neurons types designed V0 [13], V1 [14], [15], V2 [16], [17] and V3 [18] neurons. Although these studies have provided a wealth of detail about the anatomical location, axonal projections and biophysical properties of constituents of these diverse cell types, they have not elucidated the global anatomical distribution of these functional subgroups.

In this study, we investigated the general anatomical organization of flexor and extensor interneuronal circuits within the lumbar SC. The initial theory, based on the reciprocal inhibition-based “half-center” CPG model [19], hypothesizes that reciprocal activation of hindlimb flexors and extensors reflects the reciprocal inhibition between rhythmogenic interneuronal networks. This model has provided a fruitful basis for approaching the problem of locomotor generation in limbed vertebrates and has served as the basis for more complex models [20]. Consistent with these different half-center models, one study showed that networks driving flexor and extensor motoneuron pools are functionally and anatomically separated. In the mudpuppy forelimb, the elbow flexor center was localized in the C2 segment, while the elbow extensor center was localized in the C3 and C4 segment [21]. Furthermore, it was shown that the two interneuron pools could oscillate independently. In another study, commissural interneurons located in the ventral horn of L2–L3 segments and involved in left–right coordination have been shown to be anatomically and physiologically separated. Neurons in-phase with the ipsilateral L2 activity are located more ventrally than the out of phase ones [6]. Therefore, the aim of the present study was to determine whether mammalian flexor and extensor cells exhibit a rostro-caudal functional parecellation. To address this question, we performed Ca^2+^ imaging and lesion experiments using a spinal cord (SC) preparation sectioned horizontally just above the central canal. Neurons activated during 5-HT/NMDA-induced fictive locomotion were recorded optically using the Ca^2+^ indicator fluo-4 AM and the spatio-temporal activation pattern of neurons was analyzed in relation to fictive locomotion recorded from ventral roots. Electrolytic micro-lesions localized between T12 to L2 were also performed in order to detect possible selective disruption of either flexor or extensor motor output.

## Methods

### Ventral SC dissection

**Figure 1:**
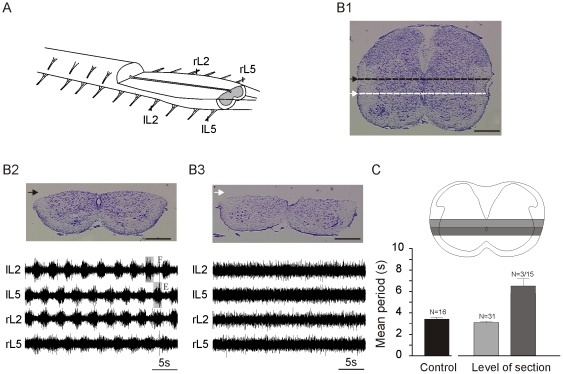
Experimental setup. A, Schematic drawing of the *in vitro* ventral SC preparation with VRs recording electrodes positioned at L2 and L5 (r = right; l = left). B, Transverse section of an intact SC (B1) and of two SC preparations with a horizontal cut at different dorso-ventral levels (B2, B3). Scale bars = 300 µm. The bottom panels show the motor activity recorded in VRs. F = Flexor activity, E = Extensor activity. C, Schematic drawing of transverse sections of the SC illustrating horizontal cuts at different levels (light grey bar and dark grey bar). The histogram plots the data of the mean period ± SEM of the different horizontal cuts.

All procedures were approved by the Animal Care and Use Committee at the University of Bordeaux and conform to the guidelines of the European Community Council (86/609/EEC). Experiments were carried out on P1 to P4 Wistar rats of both sexes (n = 88). Animals were anaesthetized by hypothermia, decapitated, eviscerated and transferred to a solution of oxygenated cold artificial cerebrospinal fluid (aCSF; pH: 7.4) of the following composition (in mM): NaCl:130, NAH_2_PO_4_:0.58, MgSO_4_:1.3, CaCl_2_:2.5, NaHCO_3_:25, D-Glucose:10, KCl:3. The experimental procedure has previously been described [22], [23]. Briefly, SCs with ventral roots (VRs) were dissected, affixed dorsal side up to a block of Sylgard (Dow-Corning, USA) and transferred to the stage of a vibratome. The cords were sectioned horizontally up to the central canal (Figure 1A). The preparation was then placed dorsal side up in the recording chamber.

### Induction and Recording of locomotor-like activity

Rhythmic locomotor-like activity was induced by perfusing the preparation with aCSF containing N-methyl-D-L-aspartate (NMDA, 14 µM) and serotonin (5-HT, 16 µM). In all experiments, left (l) and right (r) lumbar (L) VR activity (lL2, rL2, lL5 and rL5) was monitored using stainless steel electrodes insulated from the bath with Vaseline. Recordings were amplified 5000× and written to disk via a Digidata 1322 interface (Axon Instruments, CA, USA). The signals were digitized (sampling rate of 2 kHz) using Axograph software (Axon Instruments, CA, USA) and analysed using Axograph×analysis plug-ins. All drugs were purchased from Sigma-Aldrich (Oakville, Ontario).

### Ca^2+^ Imaging

The membrane-permeant Ca^2+^ indicator Fluo-4 AM (50 µg, K_d_ = 350 nM, Invitrogen) was dissolved in 25 µl of the surfactant pluronic F-127 (2 g/10 ml DMSO) and diluted in 750 µl saline for a final concentration of 60 µM [24]. A specific MRP transporter inhibitor (MK-571, 50 µM) was added to the solution in order to improve fluorescent dye uptake [25]. The ventral SC preparation was incubated in the oxygenated dye solution in the dark for 1 h.

Fluorescence images were obtained using a CCD camera (Orca ER, Hamamatsu Photonics), and illuminated using a LED (Royal Blue Lumiled, Philips). The optical signals generated by labeled cells between T12 to L5 were imaged via successive 60 s recording bouts from spatially overlapping locations using an upright microscope (Nikon Eclipse E600FN) equipped with a 10× water-immersion objective and an appropriate filter set. Optical recordings were performed at 2–5 Hz using an image-acquisition system (Openlab Software).

### Electrolytic lesions

Lesions were made by passing current (100–400 mA; 1–5 sec) through a tungsten microelectrode (WPI, Florida), to induce electrocoagulation of restricted regions between T13 to L2. In 16 experiments (out of 21 experiments), the first lesion was followed by a second lesion. Preparations were allowed to recover for 40–60 min between lesions. To confirm that the lesion was effective, transverse sections were made at the end of lesioning experiments and a numerical assessment of lesion depth was performed.

### Histology

SCs were fixed (4% paraformaldehyde; 24 hrs at 4°). They were transferred into a phosphate buffered 20% sucrose solution overnight, rinsed with PBS, frozen in isopentane (−80°C, 15 min), cut into 25 µm transverse sections, mounted on slides, and stained with Cresyl-violet. The sections were photographed using an E600 epifluorescence microscope equipped with a DXM1200 digital camera (Nikon).

### Data analysis

Ca^2+^ transients were extracted using a semi-automated method [26] developed in LabView (National Instruments, Austin, TX). Traces were subjected to high-pass filtering (τ = 10 sec); relative changes in luminance are expressed as ΔF/F, and peaks were detected using standard algorithms. Some cells were weakly activated with only a small number of Ca^2+^ peaks. Thus, we further investigated the set of the most active cells that responded with at last six Ca^2+^ peaks during episodes of locomotor activity. Cells were then classified based on their coupling strength with motor output, estimated by quantifying the variance r of phases between optical trace peaks and left L2 flexor-related motor output, analyzed using custom software (Matlab). Cells were classified as rhythmic cells with a stable relation to motor output either in phase, out of phase (0.4<r) or rhythmic cells without a stable phase relation to motor output (0.2<r<0.4).

To identify clustering of cells, images and cells from consecutive recordings along the cord in each preparation were tiled; tiled composite images from all experiments were aligned using VR locations. Density plots of pooled data were then computed using the Spatstat module of the open-source statistics package R [27].

Microlesion effects on locomotor activity were characterized in terms of 1) the duration of the lesion-induced disruption of locomotor activity; 2) the change in the motor period, 3) the change in the area of the rectified bursts and 4) the phase lag between VR activities. For each analysis, the lL2 VRs activity served as the reference trace and the phase lag corresponded to the delay (by substracting the onset of rectified rL5 or rL2 bursts to the onset of rectified lL2 bursts) over the period. The parameters were measured and averaged over 1 minute of recording. Activity recorded just before the lesion was compared to activity recorded after alternating activity resumed. Statistical analyses of phase relations were performed using the Mardia–Watson–Wheeler test (W) to compare the preferred phase vector μ and its length r (inversely proportional to phase variance) before and after lesions for each experiment. All statistical analyses were carried out using Prism software (Graphpad software, CA, USA). A Student's t-test was used to compare means between two groups. A one-way ANOVA, followed by a Tukey's test for post-hoc analysis was used to compare means between more than two groups. A confidence level of P<0.05 was considered statistically significant. Statistical values were express as means +/− SEM.

## Results

### The ventral SC preparation is sufficient to produce a well-organized locomotor activity

For our experimental paradigm, it was crucial to determine the lowest level at which an horizontal section could be performed in order to have the best spatial resolution for Ca^2+^ imaging of cells, while preserving stable rhythmic activity (Figure 1). A series of experiments was therefore carried out, in which sections were performed at different levels relative to the central canal (Figure 1A, 1B1). When the cord was transected dorsal to the central canal (n = 31; black arrow Figure 1B1, 1B2), locomotor period in the transected cord (3.1±0.1 s; light grey bar, Figure 1C) was not statistically different from that recorded in the intact spinal cord (3.4±0.2 s; black bar Figure 1C). In contrast, when the SC was transected just ventral to the central canal (white arrow Figure 1B1, 1B3) all rhythmic activity ceased (n = 12) or slowed dramatically in three cases where the sections were at the dorsal limit of the dark grey bar (period = 5.8±1.4 s; dark grey bar, Figure 1C). These results confirm that locomotor activity is preserved after a section just above the central canal [22] and shows that the spinal cord layer about 100 µm above the central canal is crucial for the generation of coordinated rhythmic activity (Figure 1C, light grey bar).

### The dorsal surface of L1 contains a higher density of locomotor rhythmic cells than the other segments

**Figure 2:**
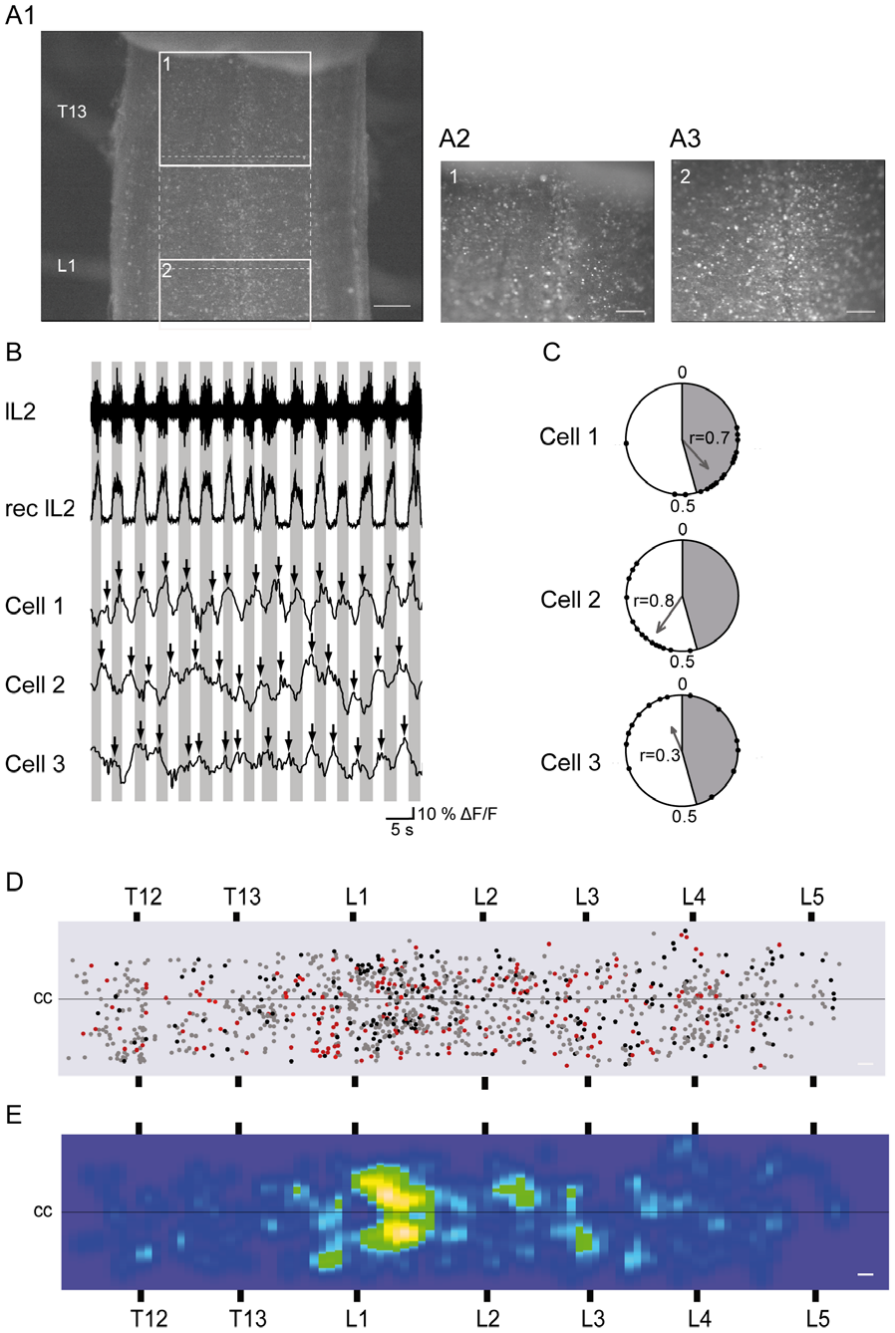
Ca^2+^ imaging experiments. A, Photomicrographs showing Fluo-4 AM labeling in the *in vitro* ventral SC preparation at the T13-L1 level (A1, magnification ×4; scale bar = 200 µm). Right photomicrographs were taken from areas delineated by grey squares 1 and 2 (A2, A3, magnification ×10; Scale bar = 100 µm). B, Extracellular recordings of left L2 VR and its rectified and low-pass filtered trace illustrating a typical rhythmic activity. Grey bars are aligned with r L2 VR bursts and reveal three cells examples tending to have Ca^2+^ peaks in phase (cell1), out of phase (cell2) or mixed (cell3) with lL2 VR activity. C, Circular plots illustrating the Ca^2+^ peak phases (black circles) for each cell in relation to the timing of L2 VRs bursts. The mean VR bursts over a 60 s recording bout range from spans phases 0 to 0.4 in the circular plot and is illustrated in grey. Vectors show the mean phase values and r values. Vector orientation indicates preferred phase of firing; vector length is proportional to coupling strength. D, Dot diagram shows distribution of all cells with phase relationship with motor output from T12 to L5. Cells were illustrated in phase (red dots), out of phase (black dots) and mixed (grey dots). E, Density plot of in phase and out of phase cells from T12 to L5 segments (n = 4 experiments; cc: central canal; scale bar = 100 µm).

We then performed series of Ca^2+^ imaging experiments (n = 4). Successive optical recordings were performed to characterize locomotor cells in the region between segments T12 and L5, during fictive locomotion induced by NMDA/5-HT application (Figure 2A). Cells were classified in phase (cell 1, Figure 2B, C; r>0.4), out of phase (cell 2, Figure 2B, C; r>0.4) or mixed cells (cell 3, Figure 2B, C; 0.2>r>0.4).

Because no extensor or flexor cluster were observed in individual optical recording datasets, data across experiments were combined using VR location for alignment. The dot diagram of these pooled data reveals that in phase (Figure 2D, black circles) and out of phase cells (Figure 2D, red circles) were interdigitated along the SC and did not reveal a preferential clustering for flexor or extensor cells as reported in mudpuppy (see Discussion). Nevertheless, a density plot generated from all cells correlated with locomotor cycle reveals that they were concentrated at the L1 segment of the SC (Figure 2E).

### L1 segment lesions elicit greater locomotor activity disruption than lesions to neighboring segments

**Figure 3:**
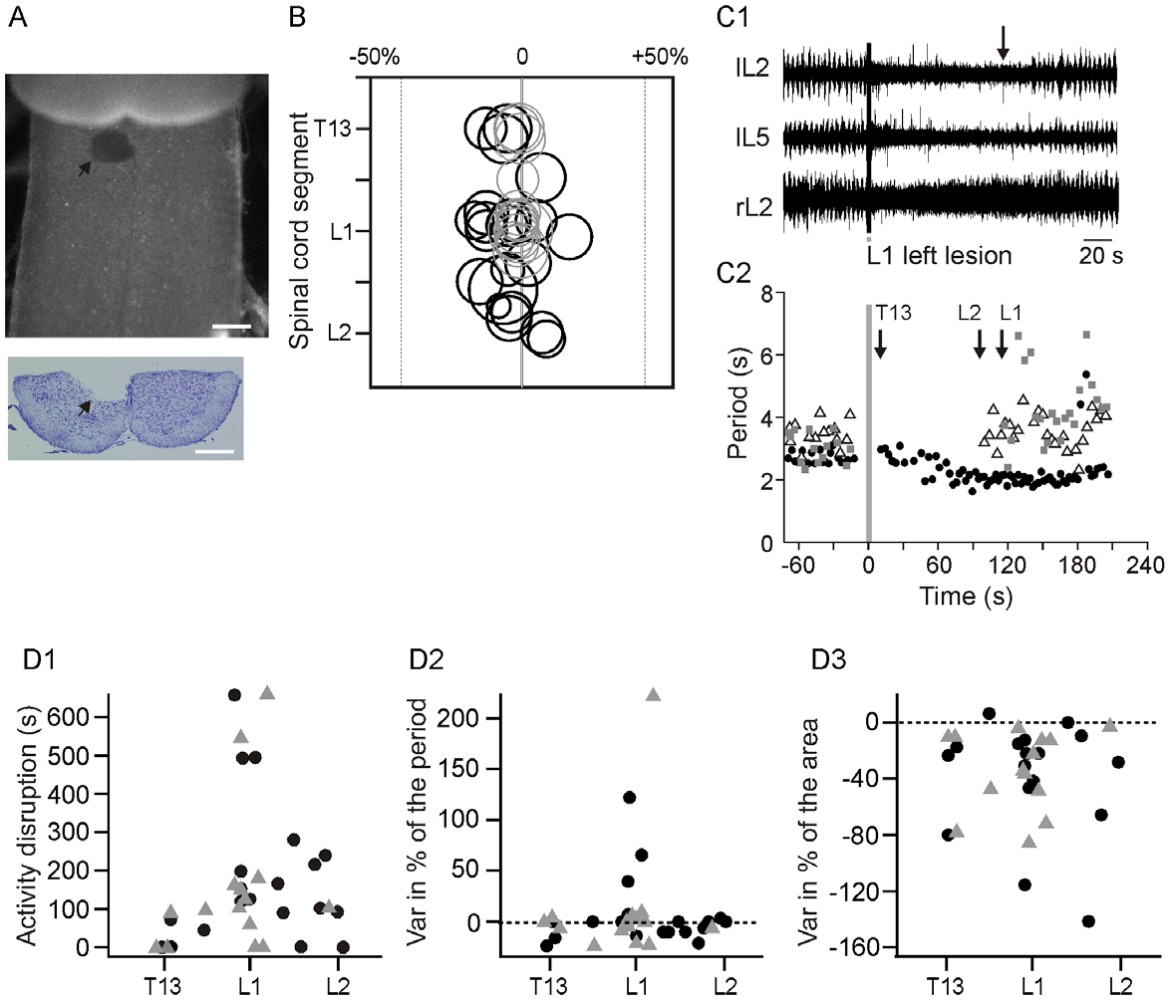
Lesion experiments. A, Photomicrographs of a ventral SC preparation (top) and its corresponding transverse section (bottom) showing an example of electrocoagulation of a restricted region located at the T13 segment level (magnification 4×; scale bar = 300 µm). B, Schematic drawing presenting all microlesions performed from T12 to L2 segments. The thick circles represent the first lesion (n = 21) whereas the thin circles represent the second lesion n = 16/21). The right and left dashed lines delineate the grey and white matter. The lesion size is expressed as a percentage of SC size (central canal set to 0). C, Lesion effects on locomotor activity. (C1), Extracellular recordings of L2 and L5 VRs before and after a lesion at the L1 level. The arrow represents the onset of locomotor recovery. (C2), Time course of the motor period 1 min before (control) and after a lesion at the T13 level (circles), the L1 level (triangles) and L2 level (squares). The onset of recovery is indicated by successive arrows for each lesion. D, Lesion effect on duration of locomotor activity disruption (D1), motor period (D2), and burst area (D3) of microlesions performed at different SC level (T13-L1-L2). The black circles indicate the first microlesions and the grey triangles illustrate the second microlesions.

To test whether anatomical segregation of neurons controlling flexor and extensor-related-activity exist more deeply in the tissue, we performed microlesion experiments (n = 21; Figure 3A) and investigated whether localized lesions induced selective disruption of either flexor or extensor motor output. Figure 3B illustrates the rosto-caudal and the medio-lateral location of the lesions. The size of the lesions (thick black circles = first lesion, thin black circles = second lesion, mean diameter = 208±6 µm) is expressed as a percentage of each SC thickness. The mean lesion depth measured in all spinal cord was 329±26 µm. The lesions' effect on locomotor activity was dependent on lesion location (Figure 3C and D). Typical examples of the effect of different lesions on the L2 motor period are illustrated in Figure 3C. The arrows indicate the recovery of locomotor activity for each lesion (T13, L2 and L1 lesion). A lesion performed at the T13 segment induced a short disruption of activity (mean = 29.3±17.8 sec, n = 4, Figure 3C2 black arrow, and Figure 3D1, T13 level). In contrast, lesions performed at the L1 and L2 segments blocked the locomotor activity for a longer period of time (Figure 3D1, L1 mean = 273.3±71.4 sec, n = 9; L2 mean = 137.3±37.5 sec, n = 8). The period and area of L2 VRs bursts were measured at the onset of the alternating activity recovery (Figure 3D2 and D3). In all cases, no change or a slight acceleration of the locomotor rhythm was observed following lesions at the T13 (mean = −10.4±6.2% of the output motor period, n = 4) and L2 level (mean = −3.1±3.4% of the output motor period, n = 8; Figure 3D2). In contrast, in 5 out of 9 cases of lesions at the L1 level induced a short or a long-lasting increase in locomotor period (mean = +41.2±20.6%) whereas the other 4 cases induced nothing or a slight acceleration of the motor period (mean = −6±3.6%). For all levels of lesion, burst area was reduced (mean = −27.6±18.5% for T13 lesions; mean = −37.3±11.9% for L1 lesions and mean = −48.2±25.9% for L2 lesions; Figure 3D3). Furthermore, there was no relationship between the size of the lesion and i) the duration of locomotor disruption ii) the motor period, and iii) the burst area (one-way ANOVA, p>0.05). Interestingly, the second lesion did not induce a greater effect than the first lesion on locomotor activity (grey triangles, Figure 3D1–3).

In the *in vitro* SC right and left side alternation is present at all segmental levels; further, alternation between left L2 and L5 provides access to flexor and extensor phase activity. Therefore, we analyzed the phase-lag between lL2 and rL2 and between lL2 and lL5 to determine if there was a change in the flexor/extensor alternation (data not shown). Following lesion experiments, the mean phase values remained unchanged (from 0.6 to 0.4) indicating that the lL2/rL2 and lL2/lL5 motor bursts remained in antiphase after the different lesions. Whatever the level of the lesion, the mean of the phase-lag was not statistically significant.

Overall, results show that L1 segment lesioning induces the most important changes in the locomotor like activity but that localized lesions between T12 to L2 segments do not lead to selective disruption of either flexor or extensor motor output.

## Discussion

During the last decade, Ca^2+^ imaging techniques have become a powerful tool for analyzing the patterns of activity of neuronal populations. In mammals, the majority of previous Ca^2+^ imaging studies in the SC focused on the activity of specific populations of spinal neurons such as motoneurons [28], identified HB9 interneurons [29] or V2a interneurons [30]. Only one recent study examined the activity of networks of heterogeneous interneurons in the SC [31]. Nevertheless in contrast to our work, this study used high magnification to record the somatic Ca^2+^ transients of the cells located medially near the midline and the analysis was limited to the upper lumbar segment (T13-L2). Therefore, this study did not provide a general mapping view of the overall SC. In contrast, to determine whether a functional-anatomical organization exists, parcellating interneurons controlling left/right alternation or flexor and extensor activity between T12 and L5, it was necessary for us to monitor a larger field of view. For this purpose, we used low magnification to detect, in the same focal plane, the phase preferences of the maximum number of spinal cells located not only near the midline, but also more laterally. This experimental procedure allowed for the first time to estimate the full neuronal activity along the entire lumbar SC (Figure 2D).

Several types of loading techniques have been previously used to label the spinal cells. Retrograde loading of Ca^2+^ green dextran amines has been used to label motoneurons and detect the spatiotemporal organization of their recruitment during locomotor-like activity [28]. Electroporation has also been used to load neurons from L5 to S2 with Ca^2+^ sensitive dyes in the isolated SC of the neonatal mouse *in vitro* [32], whereas pressure ejection of the dye between T13 and L2 segments has been performed to detect the activity of interneurons during locomotion [29], [31]. In the present study, all cells at or near the surface of the cord were loaded with membrane-permeable Ca^2+^ indicators to detect the Ca^2+^ transients from heterogeneous cells at the preparation's surface. This loading method together with automated detection of cellular activity was shown to be an efficient procedure to measure cellular activity at the system level in the respiratory network [24], [26]. This method has the great advantage of easily resolving the activity of individual neurons with epifluorescence microscopy.

Because fluo-4 AM is known to label both neuronal and glial populations, it cannot be ruled out that some of the signals may be of glial origin. Nevertheless, in neurons, Ca^2+^ influx accompanying action potentials is rapid and due to influx through high voltage-activated channels, while Ca^2+^ influx in glia is typically slower [33]. Spontaneous astrocytic Ca^2+^ oscillations in situ drive NMDAR-mediated neuronal excitation, thus based on the steep rise associated with Ca^2+^ signals recorded here, it is likely that they are of neuronal origin [33].

In all experiments of this study, care was taken to rule out the possibility that the difference of the concentration of neurons in L1 was due to an experimental bias: first, we verified that the cut was uniform at all the spinal cord levels by performing transverse sections from T13 to L5. Second, in contrast to local dye application methods used by others [31], [32], all networks from T13 to L5 were uniformly labeled by bath-application of the membrane permeant indicator. Thus, the observation of locomotor-related activity concentrated at L1 likely indicates functional specialization of networks in this region.

Although optical recordings revealed a higher density of locomotion-modulated interneurons at L1, lesions to this region did not eliminate fictive locomotion. L1 lesions typically elicited a longer lasting disruption of locomotor output, slowed locomotor period, and decreased VR burst amplitude, but in all cases, alternating, coordinated locomotor output returned. These results support two hypotheses: either networks mediating flexor-extensor coordination were outside the lesion sites, or multiple mechanisms give rise to flexor-extensor coordination such that a lesion to one network led to compensatory activation of the other. These findings are consistent with a wide range of studies that reveal the robustness of motor patterns to focal lesion in classic “labeled line” systems [34], and to both coarse and focal lesions to networks in ventrolateral medulla that generate respiratory rhythm [35], [36] as well as a classic study characterizing the robustness of memory to lesion [37]. Thus this characteristic of robustness to lesion appears to be a generic feature of vertebrate neuronal networks. It follows from this that locomotion arises out of the activity of a dispersed, heterogeneous network, capable of maintaining locomotion so long as any part of it is preserved. In the case of the respiratory CPG, one region, the pre-Bötzinger Complex (PreBötC), has been proposed as the kernel for respiratory rhythm generation, and indeed, a highly specific acute inactivation of subsets of PreBötC neurons leads to fatal apnea in intact rats [38], but larger lesions, administered over weeks to the same structures, had no effect on breathing in intact goats [36]. These findings provide support for a functional organization in which functionally similar, anatomically and/or mechanistically distinct networks coexist [39] and can be recruited to generate a qualitatively similar if other networks are inactivated [40] or lesioned, as is the case here. Consitent with this interpretation is the persistence of locomotion in all null mutants that have thus far been generated, which lack specific locomotor interneuron phenotypes [9]. Thus, the functional specialization inferred from the high numbers of locomotion-modulated interneurons in T13-L1 is not necessary incompatible with the robustness of locomotor rhythm generation to lesions in this region.

Little is known about the network structure of the mammalian locomotor CPG. The conceptual organization of the CPG for walking has been strongly influenced, however, by the half-center model of Brown (1911), developed to account for the alternating activation of flexor and extensor muscles in the cat during walking. In this model, each pool of motoneurons for flexor or extensor muscles is driven by a corresponding half center of interneurons suggesting a spatial organization of the half centers. In the mudpuppy, this parcellation has been proven because the neuronal networks for forelimb rhythmic flexor and extensor activation have been localized in two separate segments of the SC (C2 for flexor and C3 for extensor, [21]). Here, optical recording data support the conjecture that in rats, locomotor interneurons recorded at the exposed surface of the transected cord are not physically segregated based on phase of activity. The fact that the ventromedial population of commissural interneurons exhibit some degree of anatomical separation [6] could suggest that segregation of interneurons may ocur in the deeper ventral region of the spinal cord. Given the size of the lesions (329 µm of depth) in our study, these commissural interneurons were likely ablated. Nonetheless, left/right alternation remain unchanged and no selective loss of flexor or extensor motor output was observed. A complete description of locomotor networks will require true 4-dimensional data that are beginning to become available using 2-photon techniques, which might reveal a helical or braided parcellation of flexors and extensors. However, it is unlikely that our methods would have missed a simpler anatomical parcellation: due to both experimental and biological variability, locomotor interneuron networks were sampled over a range of ventrodorsal levels of section. Despite this, neither in the individual datasets, nor in the aggregated data, were flexors and extensors found to be parcellated.

By combining optical recordings with focal lesions, a more detailed description of the hindlimb locomotor CPG emerge: locomotion-modulated interneurons are more concentrated at L1. Neither in individual optical recording experiments, nor in data pooled across experiments was there evidence for spatial parcellation of flexor and extensor pools. This lack of anatomical segregation was corroborated by the lesion studies, which in all cases failed to disrupt flexor-extensor alternation. Taken together, these findings suggest that locomotion arises out of the activity of a spatially dispersed, functionally redundant network, whose coordination is mediated by interdigitated flexor and extensor interneurons. Confirmation of these findings awaits simultaneous recordings of locomotor interneurons at various depths in the cord.
